# Isolated Tuberculous Transverse Myelitis Without Meningitis Among Patients With AIDS: A Case Report

**DOI:** 10.7759/cureus.50650

**Published:** 2023-12-17

**Authors:** Kirun Angkanavisan, Minth Punpichet, Worapong Nasomsong

**Affiliations:** 1 Division of Infectious Disease, Department of Internal Medicine, Phramongkutklao Hospital and College of Medicine, Bangkok, THA; 2 Department of Radiology, Phramongkutklao Hospital and College of Medicine, Bangkok, THA

**Keywords:** spinal tuberculosis, mycobacterium tuberculosis, aids, transverse myelitis, tuberculosis

## Abstract

Acute transverse myelitis is an inflammatory disorder of the spinal cord, characterized by acute or subacute onset of paraparesis, bilateral sensory deficit, and impaired urinary bladder and sphincter tone function. *Mycobacterium tuberculosis*, a very rare cause of transverse myelitis, especially tuberculous myelitis without meningitis, is extremely rare. The main etiologic mechanism consists of an abnormal activation of the immune system against the spinal cord as well as the direct invasion by the bacillus. We present a 30-year-old Thai woman with AIDS, presenting with paraplegia for two days. Her MRI of the whole spine showed nodular enhancing intramedullary lesions involving the spinal cord at the T11-T12 level, and intramedullary enhancing lesion along the T12 spinal cord to the conus medullaris. Cerebrospinal fluid (CSF) examination revealed only a few white blood cells without hypoglycorrhachia or elevated CSF protein. CSF polymerase chain reaction (PCR) and culture for *M. tuberculosis* produced negative results. Other investigations did not demonstrate other organ involvement. Spinal cord biopsy at T12 was performed and exhibited diffuse epithelioid histiocytic proliferation admixed with small lymphocytes and plasma cells with numerous acid-fast bacilli (AFB)-positive bacilli organisms. PCR for *M. tuberculosis* was also detected in spinal cord tissue. Thus, acute transverse myelitis caused by isolated tuberculous myelitis without meningeal involvement was diagnosed. She had marked clinical improvement and neurologic recovery after treatment with anti-tuberculosis and intravenous steroid pulses. Isolated *M. tuberculosis *spinal tuberculous myelitis without meningitis is exceptionally uncommon and should be carefully considered, particularly in severely immunocompromised individuals residing in regions with a high tuberculosis burden.

## Introduction

Acute transverse myelitis (ATM) is an inflammatory disorder of the spinal cord, characterized by the acute onset of paraparesis, bilateral sensory deficit, impaired sphincter function, and a spinal segmental sensory level without clinical or laboratory evidence of spinal cord compression [1]. Mycobacterium tuberculosis, traditionally recognized as a rare cause of transverse myelitis, assumes an even more infrequent role in the manifestation of tuberculous myelitis without concurrent meningitis. The main etiologic mechanism is believed to be an abnormal activation of the immune system against the spinal cord. Other suspected mechanisms include the direct invasion by the bacillus and the toxic effect of anti-tuberculous drugs [2]. Diagnosis is achieved using the patient’s medical history, cerebrospinal fluid analysis, spinal cord magnetic resonance imaging, and bacteriological confirmation of tuberculous infection. One recent study reported that tuberculous myelitis mostly comprised meningitis and other organ involvement, especially in the pulmonary system. The distinguishing features differentiating tuberculous meningitis from other myelitis were markedly elevated cerebral spinal fluid (CSF) protein and spinal meningeal enhancement [3]. Hence, this distinctive form of tuberculous myelitis, occurring in isolation without meningitis, is exceptionally uncommon. Its occurrence warrants careful consideration, particularly in individuals experiencing severe immunocompromise, such as those with AIDS. The heightened susceptibility in severely immunocompromised individuals is accentuated when residing in regions burdened by a high prevalence of tuberculosis. The complexity of this clinical scenario emphasizes the importance of vigilance among healthcare professionals in recognizing atypical presentations of mycobacterial infections, especially in populations with heightened vulnerability. Understanding the rare occurrence of isolated tuberculous transverse myelitis without meningitis is pivotal for accurate diagnosis and timely intervention, contributing to improved outcomes in patient care.

Here, we report a 30-year-old woman with AIDS, presenting with isolated tuberculous transverse myelitis without meningitis and other organ involvement.

## Case presentation

**Figure 1:**
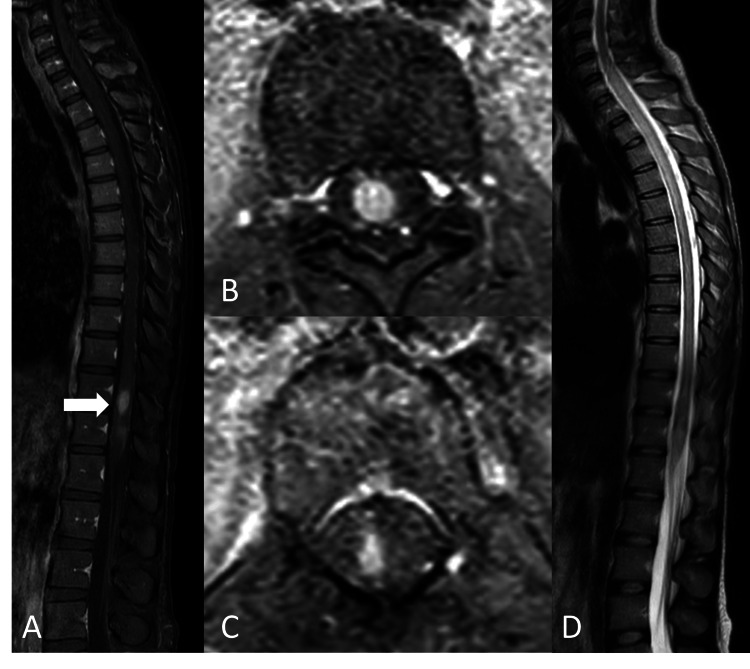
(A, B, C) Whole spine MRI with sagittal and axial planes of T1W fat suppression with gadolinium enhancement sequences demonstrating a few nodular enhancing intramedullary lesions involving the spinal cord at the T11 level to the conus medullaris about the central region (white arrow). (D) Sagittal T2W sequence showing a non-enhancing longitudinal T2 hyperintense signal change intramedullary lesion involving a central portion of the spinal cord extending from C6 to the conus medullaris.

A 30-year-old Thai woman, an office worker, HIV positive, exhibited poor adherence to antiretroviral therapy. Her last CD4 count was 15 cells/mm^3^ (2%) and her HIV-1 RNA viral load was 283,000 copies/mL. Her current medication included tenofovir/emtricitabine and lopinavir/ritonavir. She presented with paraplegia for two days. One week prior, she experienced moderate, dull-aching pain originating in the middle of her back, without any radiating pain, accompanied by a low-grade fever. The pain was unrelated to posture, and no numbness or weakness was observed. Five days prior, she complained of numbness and tingling below the umbilical area. Four days prior, she reported more numbness and decreased sensation below the umbilical area, accompanied by weakness in both lower extremities. Two days prior, all symptoms were still progressing without any improvement. She experienced difficulty urinating and constipation, and she reported neither smoking nor consuming alcohol. Neurological examinations demonstrated tenderness along the T10-L5 level without stepping, motor power graded 0 in all lower extremities, loss of pinprick, and temperature sensation below the T10 level and in the saddle area (S2-4). Also noted was a loss of proprioception and vibrational feeling, as well as a loss of gross touch in both lower extremities. Deep tendon reflexes were graded 0 in the lower extremities, and a loss of perianal sensation and sphincter tone was reported. Other examinations revealed mild pale conjunctivae and pruritic purpura eruption on all extremities. Initial laboratory results showed moderate anemia with leukopenia (Table 1). Her magnetic resonance imaging (MRI) of the whole spine showed a 0.8 x 0.8 x 1.6 cm nodular enhancing intramedullary lesion involving the spinal cord at the T11-T12 level about the central region with perilesional spinal cord edema, as well as another 0.8 x 0.4 x 1.0 cm intramedullary enhancing lesion along the T12 spinal cord to the conus medullaris (Figure 1). Lumbar puncture was performed, and the cerebrospinal fluid (CSF) profile showed a clear appearance, open pressure 7 cmH2O, WBC 9 cells/mm^3^, lymphocyte 100%, RBC 600 cells/mm^3^, protein 132 mg/dL, and sugar 48 mg/dL. CSF multiplex polymerase chain reaction (PCR) panel (BIOFIRE® FILMARRAY® Meningitis/Encephalitis [ME] Panel, bioMérieux) and GeneXpert were undetected. CSF culture for mycobacterium subsequently revealed no growth. Therefore, a spinal cord biopsy at the T12 level was performed after 17 days of admission, owing to undiagnosed myelitis.

**Figure 2:**
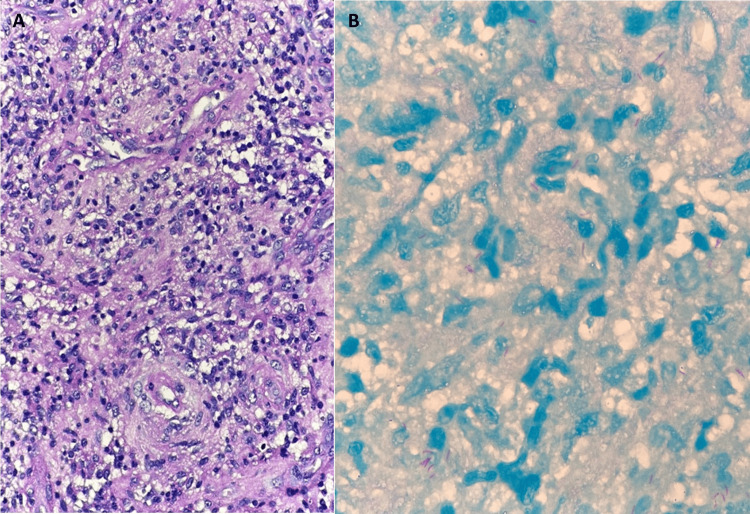
Spinal cord biopsy with H&E and AFB stain demonstrated diffuse epithelioid histiocytic proliferation admixed with small lymphocyte and plasma cells (A) with numerous AFB-positive bacilli (B). H&E: hematoxylin and eosin, AFB: acid-fast bacilli

The histopathologic findings demonstrated diffuse epithelioid histiocytic proliferation admixed with small lymphocytes and plasma cells with numerous acid-fast bacilli (AFB)-positive bacilli organisms (Figure 2). PCR for *M. tuberculosis* complex was positive in the intramedullary mass. However, spinal tissue culture for mycobacteria indicated no growth, so the antimicrobial susceptibility of M. tuberculosis in this case was not established. The computed tomography (CT) of the chest and whole abdomen revealed a 0.5-cm calcified nodule in the anterior basal segment of the left lower lung and a few calcified nodules in the subcarinal and paraesophageal regions, likely representing calcified granulomas and lymph nodes. Sputum GeneXpert and culture for mycobacterium results were both negative.

Rifampicin, pyrazinamide, ethambutol, and levofloxacin were administered as an anti-tuberculosis regimen for 12 months. The use of levofloxacin instead of isoniazid was due to the patient experiencing isoniazid-induced liver injury. Additionally, intravenous dexamethasone was combined for one week, followed by oral prednisolone in diminishing doses for three weeks.

One month after the antituberculosis treatment, the antiretroviral drugs were restarted, including tenofovir, emtricitabine, and efavirenz. The patient's symptoms significantly improved after approximately four months of treatment, and she was discharged with a partially bedridden status. During the outpatient visit, her overall symptoms remained stable, and she could walk with the assistance of ambulatory devices.

**Table 1:** Laboratory profile pf the patient. AFB: acid-fast bacillus, CSF: cerebrospinal fluid, MTB: *Mycobacterium tuberculosis*, RIF: rifampicin

Laboratory	First day of admission	Reference range
Hematocrit (%)	29.4	35.7–45.1
Hemoglobin (g/dL)	8.9	12.1–14.7
White blood cells (cell/µL)	2,700	4,400–10,800
Neutrophils (%)	68	39.2–70.8
Lymphocytes (%)	18	20–48.4
Platelets (cell/µL)	338,000	184,000–422,000
CD+ 4T cells (cell/µL/%)	10/3	470–1,404/ 24-51
Serum albumin (U/L)	4.09	3.5–5.2
Aspartate transaminase (U/L)	17	0–50
Alanine transaminase (U/L)	9	0–50
Alkaline phosphatase (U/L)	85	35–104
CSF profile		
Appearance	Clear	Clear
Open pressure (cmH_2_O)	7	7–18
White blood cell (cell/mm^3^)	9	<5
Neutrophils (%)	–	–
Lymphocytes (%)	100	–
Red blood cell (cell/mm^3^)	–	0
Protein (mg/dL)	132	15–45
Sugar (mg/dL)	48	40–70
AFB	Not seen	–
Xpert MTB/RIF	Not detected	–
Mycobacteria culture	No growth	–
Cryptococcus antigen	Negative	–

## Discussion

Tuberculous myelitis mostly affects previously healthy adolescent and young adult individuals. The clinical manifestations include fever, headache, paraparesis, altered gait, bladder and bowel dysfunction, and sensory impairment. Tuberculous myelitis commonly presents with evidence of either tuberculous meningitis or pulmonary tuberculosis, while isolated tuberculous myelitis is extremely rare [3-6]. Regarding neuroimaging, spine MRI is an essential tool in diagnosing tuberculous myelitis associated with longitudinally extensive myelitis [3, 5, 7]. The cervicothoracic dorsal spinal cord is the most commonly affected area, approximately 90%, with hyperintensity on T2-weighted images being the most consistent finding [4, 5]. Apart from myelitis, the MRI of the spine showed other common features consisting of spinal meningeal enhancement (53-77.8%), extra-axial collection (16.7%), CSF loculation (5-16.7%), arachnoiditis (16.7-21%), and spinal tuberculoma (11.1-32%) [3, 8]. An MRI of the brain also showed hydrocephalus (38.9%), basal exudates (44.4%), meningeal enhancement (61.1%), tuberculomas (50%), and infarct (11.1%). CSF GeneXpert MTB/RIF was positive at 16.7%, and the CSF examination usually exhibited elevation of protein, low glucose, and pleocytosis [3, 8]. This case demonstrates an exceptionally rare manifestation of TB myelitis without involving other organs, particularly the meninges. Hence, the abnormal calcified lesion in the chest CT is probably caused by old pulmonary tuberculosis. A key strength of this case report is the use of a spinal biopsy, revealing numerous bacilli in the spinal tissue and confirming the pathophysiology related to the direct invasion of bacilli. Quite probably, acute transverse myelitis in this case is caused by direct invasion, representing a highly uncommon mechanism.

Several studies have indicated the potential effectiveness of combining steroid and antituberculosis treatments for TB myelitis. Clinical improvements, on average, materialized approximately 12 weeks after the initiation of treatment [3, 8, 9]. However, a substantial proportion of cases (42.3%) experienced poor outcomes, with a significant number enduring severe neurological deficits, while others demonstrated near-complete or partial recovery [3, 8]. The critical importance of timely diagnosis and treatment, particularly before the onset of permanent neurological deficits, was underscored. In instances involving patients with HIV, the treatment protocol may integrate antiretroviral therapy. Despite a definitive diagnosis of TB myelitis and the administration of appropriate treatment, a unique and rarely encountered manifestation led to irreversible neurological deficits in the examined case. The delayed definitive diagnosis and treatment were identified as contributing factors to this unfortunate outcome. In cases of acute transverse myelitis, tuberculosis can swiftly progress, potentially resulting in enduring disability.

In patients presenting with acute transverse myelitis, tuberculosis can rapidly progress, potentially leading to permanent disability. Tuberculosis should be considered as a potential etiologic agent, especially in severely immunocompromised patients, even when CSF examination yields normal results or there is no apparent pulmonary involvement. Consequently, the diagnosis of this condition can be challenging, necessitating a prompt and invasive tissue biopsy when initial findings prove inconclusive. Timely and appropriate diagnosis and treatment remain pivotal for achieving more favorable outcomes.

## Conclusions

*Mycobacterium tuberculosis* has the capacity to induce transverse myelitis by directly invading the spinal cord, resulting in isolated spinal tuberculous myelitis. This particular form of myelitis is exceptionally rare and demands careful consideration, especially in severely immunocompromised individuals residing in regions burdened by a high prevalence of tuberculosis.
